# Safety and efficacy of stent-assisted coiling for acutely ruptured wide-necked intracranial aneurysms: comparison of LVIS stents with laser-cut stents

**DOI:** 10.1186/s41016-021-00237-1

**Published:** 2021-03-03

**Authors:** Gaici Xue, Qiao Zuo, Xiaoxi Zhang, Haishuang Tang, Rui Zhao, Qiang Li, Yibin Fang, Pengfei Yang, Bo Hong, Yi Xu, Qinghai Huang, Jianmin Liu

**Affiliations:** 1 Department of Neurosurgery, General Hospital of Southern Theatre Command of PLA, 111 Liuhua Road, Guangzhou, 510010 China; 2 Department of Neurosurgery, Changhai Hospital, Naval Medical University, 168 Changhai Road, Shanghai, 200433 China

**Keywords:** Ruptured intracranial aneurysm, LVIS stent, Laser-cut stent, Propensity score matching

## Abstract

**Background:**

To compare the safety and efficacy of LVIS stent-assisted coiling with those of laser-cut stent-assisted coiling for the treatment of acutely ruptured wide-necked intracranial aneurysms.

**Methods:**

Patients with acutely ruptured wide-necked intracranial aneurysms treated with LVIS stent-assisted coiling (LVIS stent group) and laser-cut stent-assisted coiling (laser-cut stent group) were retrospectively reviewed from January 2014 to December 2017. Propensity score matching was used to adjust for potential differences in age, sex, aneurysm location, aneurysm size, neck width, Hunt-Hess grade, and modified Fisher grade. Perioperative procedure-related complications and clinical and angiographic follow-up outcomes were compared. Univariate and multivariate analyses were performed to determine the associations between procedure-related complications and potential risk factors.

**Results:**

A total of 142 patients who underwent LVIS stent-assisted coiling and 93 patients who underwent laser-cut stent-assisted coiling were enrolled after 1:2 propensity score matching. The angiographic follow-up outcomes showed that the LVIS stent group had a slightly higher complete occlusion rate and lower recurrence rate than the laser-cut stent group (92.7% vs 80.6%; 3.7% vs 9.7%, *P* = 0.078). The clinical outcomes at discharge and follow-up between the two groups demonstrated no significant differences (*P* = 0.495 and *P* = 0.875, respectively). The rates of intraprocedural thrombosis, postprocedural thrombosis, postoperative early rebleeding, and procedure-related death were 0.7% (1/142), 1.4% (2/142), 2.8% (4/142), and 2.1% (3/142) in the LVIS stent group, respectively, and 4.3% (4/93), 2.2% (2/93), 1.1% (1/93), and 3.2% (3/93) in the laser-cut stent group, respectively (*P* = 0.082, 0.649, 0.651, and 0.683). Nevertheless, the rates of overall procedure-related complications and intraprocedural rupture in the LVIS stent group were significantly lower than those in the laser-cut stent group (5.6% vs 14.0%, *P* = 0.028; 0.7% vs 6.5%, *P* = 0.016). Multivariate analysis showed that laser-cut stent-assisted coiling was an independent predictor for overall procedure-related complications (OR = 2.727, *P* = 0.037); a history of diabetes (OR = 7.275, *P* = 0.027) and other cerebrovascular diseases (OR = 8.083, *P* = 0.022) were independent predictors for ischemic complications, whereas none of the factors were predictors for hemorrhagic complications.

**Conclusions:**

Compared with laser-cut stent-assisted coiling, LVIS stent-assisted coiling for the treatment of acutely ruptured wide-necked intracranial aneurysms could reduce the rates of overall procedure-related complications and intraprocedural rupture.

## Background

Endovascular treatment has become an important treatment modality for the treatment of intracranial aneurysms. The use of intracranial stents has significantly broadened the indications for endovascular treatment. Stents not only provide a mechanical barrier to prevent coil protrusion into the parent artery but also, more importantly, change the hemodynamics of the parent artery and decrease the flow stress to the aneurysm neck, which promotes progressive aneurysmal thrombosis and healing of the aneurysm neck [1–4]. A growing number of studies have corroborated that stent-assisted coiling (SAC) improved the long-term outcomes of unruptured wide-necked intracranial aneurysms compared with balloon-assisted coiling or coiling only, without significantly increasing the risk of perioperative procedure-related complications [5–8].

However, for acutely ruptured intracranial aneurysms (RIAs), the perioperative safety of stent placement has been highly controversial [9–11]. The results of different studies have shown great heterogeneity, and most studies suggested that SAC for RIAs increased the incidence of hemorrhagic and ischemic events compared with non-SAC [12–17]. However, several recent studies have shown that SAC did not increase the risk of perioperative procedure-related complications for the treatment of selected wide-necked acutely RIAs [18–22]. The inconsistency of the results of these studies may be substantially attributed to the differences in periprocedural antiplatelet medication management, types of stents, operator experience and skills, and criteria for the selection of cases [6].

The low-profile visualized intraluminal support (LVIS) device (MicroVention, Tustin, CA, USA) is a self-expandable braided stent designed to have higher metal coverage and smaller cells than laser-cut stents (Enterprise, Neuroform stents, Solitaire stent, etc.) [23–25]. Several studies on unruptured intracranial aneurysms indicated that LVIS stents were associated with slightly better perioperative safety, a higher long-term complete occlusion rate and a lower recurrence rate than laser-cut stents [24–30]. However, whether the safety and efficacy of LVIS SAC in the treatment of acutely RIA are superior to laser-cut SAC is not yet clear. Therefore, we present herein a propensity score-matched cohort study to compare the safety and efficacy of LVIS SAC with laser-cut SAC for the treatment of acutely ruptured wide-necked intracranial aneurysms.

## Methods

The local institutional review board approved the study protocol, and the requirement for written informed consent was waived given the retrospective nature of the analysis.

### Patient selection and population

The inclusion criteria for this study were as follows: (1) RIA diagnosed by the combination of CT, lumbar puncture, and digital subtraction angiography; (2) aneurysm treated no more than 28 days after the initial rupture; (3) aneurysm treated by SAC; and (4) saccular aneurysm with a wide neck (neck > 4 mm and/or dome-to-neck ratio ≤ 2).

The exclusion criteria were as follows: (1) traumatic, pseudo, dissecting, fusiform, and blood blister-like aneurysms; (2) multiple aneurysms but failure to identify the ruptured aneurysm; (3) staged stent placement; (4) aneurysm was treated in other hospitals; and (5) incomplete clinical and angiographic data.

**Fig. 1:**
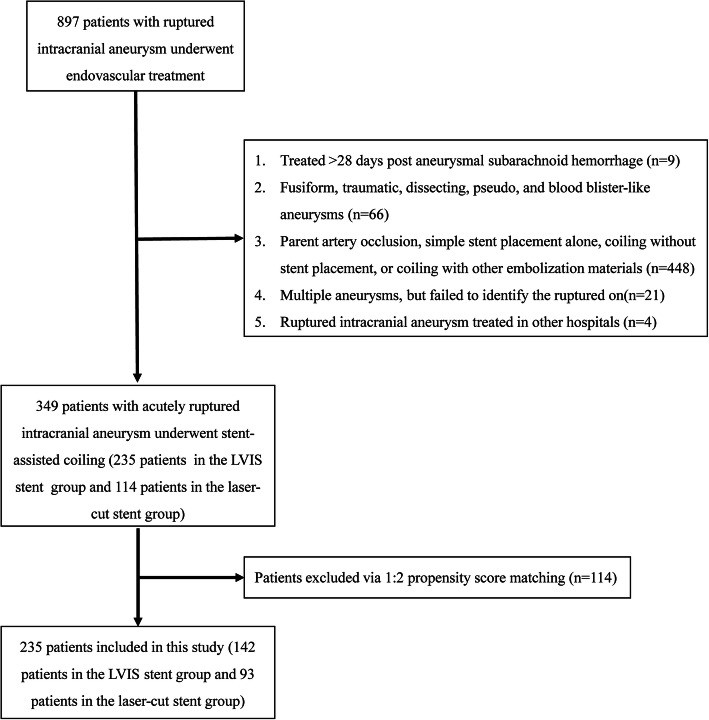
Flowchart showing the patient selection process according to the inclusion and exclusion criteria

According to the inclusion and exclusion criteria, the clinical and angiographic data of 349 patients with wide-necked acutely RIA treated with SAC were retrospectively reviewed by 2 experienced neurologists between January 2014 and December 2017, including 235 patients treated with LVIS stent-assisted coiling (LVIS stent group) and 114 patients treated with laser-cut stent-assisted coiling (laser-cut stent group). Propensity score matching (1:2 matching) was used to address potential biases in sex, age, aneurysm size, aneurysm location, neck size, Hunt-Hess grade, and modified Fisher grade between the two groups [31]. Finally, 235 patients with wide-necked acutely RIA treated with SAC were included in this study (142 patients in the LVIS stent group and 93 patients in the laser-cut stent group) (Fig. 1).

### Procedure technique, perioperative anticoagulant treatment, and antiplatelet regimens

All procedures were performed via the femoral approach under general anesthesia. Systemic heparinization was performed after femoral sheath placement to maintain an activated clotting time 2 to 3 times the baseline value during the procedure. A 6F guiding catheter was placed in the distal internal carotid artery or vertebral artery. Three-dimensional reconstruction was performed to measure the aneurysm size and neck width. All stents (LVIS, MicroVention Terumo, USA; Enterprise, Cordis, USA; Solitaire, Covidien, USA; Neuroform, Boston Scientific, USA) and coils were deployed according to the standard procedure recommended by the manufacturer. Heparin was neutralized at the end of the procedure for all patients.

A loading dose of aspirin (300 mg) and clopidogrel (300 mg) was given rectally or orally by gastric tube when the decision to perform SAC was made. A loading dose (5 μg/kg for 3 min) of glycoprotein IIb/IIIa inhibitor (tirofiban; Grand Pharma, China) was intravenously injected to prevent platelet aggregation before stent release and maintained at a rate of 0.075 μg/kg/min for 6 h. Dual antiplatelet therapy (aspirin 100 mg/day and clopidogrel 75 mg/day) was routinely administered after the operation. Clopidogrel was discontinued 6 weeks later, and aspirin (100 mg/day) was continued for the patients’ lifetime.

If acute thrombosis occurred during the procedure, tirofiban was administered through the intra-arterial microcatheter at a rate of 0.075 μg/kg/min. If aneurysm rupture occurred during the procedure, protamine sulfate was used immediately to neutralize heparin, and the coils were quickly packed for dense embolization of the aneurysm. If necessary, a balloon was used to temporarily block the parent artery to control bleeding.

In addition, according to the patients’ clinical condition, surgical procedures, including external ventricular drainage, ventriculoperitoneal shunt placement and other surgical procedures (decompressive craniectomy and/or hematoma evacuation), were performed.

### Clinical and angiographic follow-up

All patients underwent the first clinical assessment at discharge, and all patients who survived were advised to undergo clinical and angiographic follow-up at 3, 6, and 12 months after discharge and annually thereafter. Clinical follow-up was usually performed in form of outpatient clinic evaluations or telephone interviews, and the results were assessed using the modified Rankin Scale (mRS). Favorable clinical outcomes were defined as an mRS score of 0–2, and poor clinical outcomes were defined as an mRS score of 3–6. Angiographic follow-up was usually performed by magnetic resonance angiography (MRA) or DSA. Immediate embolization results were assessed using the Raymond-Roy occlusion classification, and the follow-up results were classified into four categories when compared with the immediate embolization results: (1) complete occlusion, defined as a 100% aneurysmal obliteration; (2) improvement, defined as decreased contrast material filling into the aneurysm sac; (3) stability, defined as unchanged contrast material filling into the aneurysm sac; or (4) recurrence, defined as increased contrast material filling into the aneurysm sac [22].

### Statistical analysis

All data were statistically analyzed using IBM SPSS software (version 22.0, Chicago, IL, USA). Propensity score matching in a 1:2 ratio was performed with a logistic regression model to adjust for potential biases in sex, age, aneurysm location, aneurysm size, neck size, Hunt-Hess grade, and modified Fisher grade between the two groups. Nearest neighbor matching and non-replacement sampling were used with a caliper distance of 0.1 and matching order of random. The independent samples *t* test, nonparametric test, Pearson *χ*2 test or Fisher exact test were used to analyze the matched data, as appropriate. Categorical variables are presented as numbers and percentages, and continuous variables are presented as the mean ± standard deviation (*x* ± *s*). Univariate analysis and logistic regression analysis were used to determine the independent associations between perioperative procedure-related complications and potential risk factors. Factors with *P* values < 0.10 in univariate analysis were included in the logistic regression analysis using the entry method, with an inclusion criterion of 0.05 and an exclusion criterion of 0.10. *P* values < 0.05 were considered statistically significant.

## Results

### Baseline characteristics of the patients

There were no statistically significant differences in any baseline characteristics between the two groups. Of the 235 patients, 164 (69.8%) were females. The mean patient age, aneurysm size, and neck size were 59.5 ± 12.3 years (range, 16 to 88 years), 5.1 ± 2.6 mm (range, 0.9 to 17.8 mm), and 3.6 ± 1.5 mm (range, 0.6 to 9.6 mm), respectively. A total of 211 (89.8%) aneurysms were located in the anterior circulation (Table 1).

**Table 1:** Patient and aneurysm characteristics

Variable	LVIS stent group (*n* = 142)	Laser-cut stent group (*n* = 93)	*P* value
Age, years	59.1 ± 12.0	60.0 ± 12.8	0.560
Female	99(69.7)	65(69.9)	0.977
Aneurysm size, mm	5.0 ± 2.6	5.1 ± 2.6	0.827
Neck size, mm	3.5 ± 1.5	3.7 ± 1.4	0.284
Dome-to-neck ratio	1.5 ± 0.4	1.4 ± 0.5	0.178
Hypertension	81(57.0)	57(61.3)	0.518
Diabetes mellitus	15(10.6)	8(8.6)	0.621
Coronary heart disease	7(4.9)	7(7.5)	0.411
Smoking history	14(9.9)	9(9.7)	0.963
pulmonary infection	12(8.5)	9(9.7)	0.747
Location			
Internal carotid artery	21(14.8)	14(15.1)	0.859
Middle cerebral artery	15(10.6)	8(8.6)	
Anterior communicating artery	29(20.4)	15(16.1)	
Posterior communicating artery	62(43.7)	47(50.5)	
Posterior circulation	15(10.6)	9(9.7)	
Irregular shape	71(50.0)	45(48.4)	
Bifurcation	94(66.2)	60(64.5)	0.791
Multiple aneurysms	34(23.9)	27(29.0)	0.384
Hunt-Hess grade			
I	83(58.5)	46(49.5)	0.368
II	36(25.4)	29(31.2)	
III	14(9.9)	14(15.1)	
IV	9(6.3)	4(4.3)	
Modified Fisher grade			
1	32(22.5)	16(17.2)	0.818
2	85(59.9)	59(63.4)	
3	22(15.5)	16(17.2)	
4	3(2.1)	2(2.2)	
Treatment timing			
≤ 3 days	100(70.4)	59(63.4)	0.403
3–14 days	38(26.8)	29(31.2)	
14–28 days	4(2.8)	5(5.4)	
Surgical procedure			
External ventricular drainage	6(4.2)	6(6.5)	0.548
Ventriculoperitoneal shunt	0(0.0)	1(1.1)	0.396
Decompressive craniectomy	6(4.2)	2(2.2)	0.484

Unless indicated otherwise, data are presented as the number of patients (%)

### Immediate embolization results and clinical outcomes at discharge

Immediate embolization results showed that in the LVIS stent group, Raymond class I occlusion was achieved in 91 patients (64.1%), Raymond class II in 21 patients (14.8%), and Raymond class III in 30 patients (21.1%), compared with 47 patients (50.5%), 16 patients (17.2%), and 30 patients (32.3%) in the laser-cut stent group, respectively, demonstrating a statistically significant difference between the two groups (*P* = 0.039). A total of 88.0% (125/142) of patients in the LVIS stent group and 84.9% (79/93) of patients in the laser-cut stent group had favorable neurologic outcomes at discharge, but the difference between the two groups was not statistically significant (*P* = 0.495) (Table 2).

**Table 2:** Clinical and angiographic outcomes

Outcomes	LVIS stent group	laser-cut stent group	*P* value
Immediate embolization result			
Raymond I	91(64.1)	47(50.5)	**0.039**
Raymond II–III	51(35.9)	46(49.5)	
Clinical outcome at discharge			
mRS score 0–2	125(88.0)	79(84.9)	0.495
mRS score 3–6	17(12.0)	14(15.1)	
Angiographic follow-up			
Complete occlusion	101(92.7)	58(80.6)	**0.078**
Improvement	2(1.8)	5(6.9)	
Stability	2(1.8)	2(2.8)	
Recurrence	4(3.7)	7(9.7)	
Clinical follow-up*			
mRS score 0–2	114(89.1)	76(88.4)	0.875
mRS score 3–6	14(10.9)	10(11.6)	
Clinical follow-up†			
mRS score 0–2	114(83.8)	76(84.4)	0.901
mRS score 3–6	22(16.2)	14(15.6)	

*Excluding patients who died at discharge

†Including patients who died at discharge

Unless indicated otherwise, data are presented as the number of patients (%)

### Clinical and angiographic follow-up results

Of the 223 patients who survived at discharge, a total of 214 (96.0%, 214/223) patients underwent clinical follow-up (mean 1269 days). In addition, 114 patients (89.1%, 114/128) had favorable clinical outcomes in the LVIS stent group, while 76 (88.4%, 76/86) patients had favorable clinical outcomes in the laser-cut stent group (*P* = 0.875)

A total of 181 (81.2%, 181/223) patients had at least one angiographic follow-up (mean 529 days), including 109 in the LVIS stent group and 72 in the laser-cut stent group. Angiographic follow-up results showed that in the LVIS stent group, 101 patients (92.7%, 101/109) were successfully occluded, 2 patients (1.8%, 2/109) improved, 2 patients (1.8%, 2/109) were stable, and 4 patients (3.7%, 4/109) were recanalized, compared with 58 patients (80.6%, 58/72), 5 patients (6.9%, 5/72), 2 patients (2.8%, 2/72), and 7 patients (9.7%, 7/72) in the laser-cut stent group, showing no statistically significant difference between the two groups (*P* = 0.078). No delayed rebleeding or ischemic events occurred during the follow-up period (Table 2).

### Perioperative procedure-related complications and mortality

The LVIS stent group showed a lower overall perioperative procedure-related complication rate than the laser-cut stent group (5.6% vs 14%, *P* = 0.028). The rates of hemorrhagic and ischemic complications in the LVIS stent group were slightly lower than those in the laser-cut stent group, but the differences were not statistically significant (3.5% vs 7.5%, *P* = 0.227; 2.1% vs 6.5%, *P* = 0.161).

Among the hemorrhagic complications, intraprocedural rupture occurred in 1 case (0.7%) in the LVIS stent group and 6 cases (6.5%) in the laser-cut stent group, which was a significant difference between the two groups (*P* = 0.016). Postprocedural early rebleeding occurred in 4 cases (2.8%) in the LVIS stent group and 1 case (1.1%) in the laser-cut stent group, but the difference was not statistically significant (*P* = 0.651)

Regarding ischemic complications, intraprocedural thrombosis and postprocedural thrombosis occurred in 1 case (0.7%) and 2 cases (1.4%) in the LVIS stent group, compared with 4 cases (4.3%) and 2 cases (2.2%) in the laser-cut stent group (*P* = 0.082 and 0.649), respectively.

The procedure-related mortality rate was 2.1% (3/142) in the LVIS group, including 2 cases of intraprocedural aneurysm rupture and 1 case of postprocedural in-stent thrombosis, and 3.2% (3/93) in the laser-cut stent group, including 1 case of intraprocedural aneurysm rupture, 1 case of postprocedural early rebleeding, and 1 case of postprocedural in-stent thrombosis (Table 3).

**Table 3:** Perioperative procedure-related complications and mortality

Procedure-related complications	LVIS stent group (*n* = 142)	Laser-cut stent group (*n* = 93)	*P* value
Procedure-related complications	8(5.6)	13(14.0)	**0.028**
Hemorrhagic	5(3.5)	7(7.5)	0.227
Intraprocedural rupture	1(0.7)	6(6.5)	**0.016**
Postprocedural early rebleeding	4(2.8)	1(1.1)	0.651
Ischemic	3(2.1)	6(6.5)	0.161
Intraprocedural thrombosis	1(0.7)	4(4.3)	0.082
Postprocedural thrombosis	2(1.4)	2(2.2)	0.649
Procedure-related mortality	3(2.1)	3(3.2)	0.683

Unless indicated otherwise, data are presented as the number of patients (%)

### Univariate and multivariate analysis of risk factors for perioperative procedure-related complications

The following factors were included in the univariate analysis of perioperative procedure-related complications: patient age, sex, history of hypertension, smoking history, history of diabetes, history of coronary heart disease, other cerebrovascular diseases, Hunt-Hess grade, modified Fisher grade, aneurysm size, neck size, dome-to-neck ratio, aneurysm shape, aneurysm location, treatment timing, stent type, and immediate embolization results. Univariate analysis showed that modified Fisher grade (*P* = 0.091) and laser-cut stents (*P* = 0.034) were associated with overall procedure-related complications; a history of diabetes (*P* = 0.028) and other cerebrovascular diseases (*P* = 0.057) were associated with ischemic complications, whereas none of the factors were associated with hemorrhagic complications. Multivariate analysis showed that laser-cut SAC was an independent predictor of overall procedure-related complications (OR = 2.727, 95% CI 1.063–6.998; *P* = 0.037), while a history of diabetes (OR = 7.275, 95% CI 1.519–34.833; *P* = 0.027) and other cerebrovascular diseases (OR = 8.083, 95% CI 1.343–48.644; *P* = 0.022) were independent predictors for ischemic complications.

## Discussion

In this propensity score-matched cohort study, the rates of overall procedure-related complications and intraoperative aneurysm rupture were significantly lower in the LVIS stent group than in the laser-cut stent group. The angiographic follow-up results showed that the LVIS stent group had a higher occlusion rate and lower recurrence rate than the laser-cut stent group, but the difference was not statistically significant. In addition, the rates of favorable clinical outcomes at discharge and during long-term follow-up were comparable between the two groups. Multivariate analysis revealed that laser-cut SAC was an independent predictor of overall procedure-related complications. These results suggest that LVIS SAC is safer and more effective than laser-cut SAC for the treatment of wide-necked acutely RIAs.

Although there are several studies comparing the safety and short-term efficacy of SAC with non-SAC for the treatment of RIAs, the stents used in these studies were all laser-cut stents [8, 15, 18–22, 32, 33]. Of these studies, only one study reported by Fan et al. compared the perioperative procedure-related complication rate among different types of stents for RIAs in a subgroup analysis [15]. The author reported 63 cases of laser-cut SAC and 159 cases of non-SAC for the treatment of RIAs and found that the rates of intraoperative aneurysm rupture and intraoperative thrombus formation in the laser-cut SAC group were significantly higher than those in the non-SAC group (9.5% vs 3.1%, *P* = 0.048; 15.9% vs 3.8%, *P* = 0.002). In contrast, the rates of intraoperative aneurysm rupture, intraoperative thrombus formation, postoperative early rebleeding, and postoperative ischemia were 9.1% (1/11), 9.1% (1/11), 9.1% (1/11), and 27.3% (3/11) in the Neuroform/Enterprise group, respectively, compared with 9.6% (5/52), 17.3% (9/52), 0, and 13.5% (7/52) in the Solitaire group, but none of the differences were statistically significant (*P* = 1.000, 0.676, 0.175, and 0.360, respectively). Choi HH et al. reported a cohort study of 55 cases of SAC (46 Enterprise, 9 Neuroform) and 394 cases of non-SAC for the treatment of RIAs and demonstrated that the incidence of hemorrhagic events was comparable between the laser-cut SAC and non-SAC groups (9.1% vs 4.8%, *P* = 0.19), while the incidence of thromboembolic events was significantly higher in the laser-cut SAC group than in the non-SAC group (25.5% vs 12.4%, *P* = 0.01). When focusing on an analysis of the 14 cases of thromboembolic events in the laser SAC group, 12 of the 14 thromboembolic events were treated with Enterprise stents (26.1%, 12/46), and the remaining 2 were treated with Neuroform stents (22.2%, 2/9). However, the author did not specify the type of stent used for cases of hemorrhagic events in the SAC group [32]. Zhang et al. performed a systematic review of the literature on laser-cut stents for the treatment of RIAs and found that laser-cut SAC significantly increased the long-term complete occlusion rate (73.4% vs 61.0%) and decreased the recurrence rate (4.8% vs 16.6%) compared with non-SAC but laser-cut SAC carried a higher rate of periprocedural procedure-related complications (20.2% vs 13.1%) [6]. These results suggest that although laser-cut SAC of acutely RIAs can improve the long-term complete occlusion rate and reduce the recurrence rate, this method also carried a high risk of perioperative hemorrhagic and ischemic complications, and the difference in the complication rate between different laser-cut stents was not significant.

The LVIS stent has unique advantages for treating RIAs compared with laser-cut stents. First, LVIS stents have higher metal coverage (23%) and smaller mesh (1 mm) than laser-cut stents, which provides a better flow-diverting effect and greater protection across the aneurysm neck to effectively reduce the risk of coil protrusion into the parent artery [23, 26, 34]. In addition, the smaller delivery system of LVIS stents makes it easy to reach small vessels distal to the circle of Willis. Moreover, LVIS stents can be well apposed in curved vessels, which is beneficial to reducing the incidence of in-stent restenosis events [23, 24, 35]. Wu reported 32 cases of RIA treated with LVIS SAC, but no procedure-related complications were observed [36]. Similarly, Yan et al. treated 15 cases of RIAs with LVIS SAC without procedure-related complications [37]. Our propensity score-matched cohort study showed that for wide-necked acutely RIAs, LVIS SAC yielded lower rates of perioperative overall procedure-related complications and intraprocedural aneurysm rupture than laser-cut SAC (5.6% vs 14.0%, *P* = 0.028; 0.7% vs 6.5%, *P* = 0.016). These results were similar to Chen’s study, which analyzed 92 cases of SAC for unruptured middle cerebral artery aneurysms and found that LVIS SAC significantly reduced the incidence of intraoperative aneurysm rupture compared with laser-cut SAC [38]. In addition, our present results indicated that the LVIS stent group had a higher complete occlusion rate and lower recurrence rate than the laser-cut stent group (92.7% vs 80.6%; 3.7% vs 9.7%). Although the difference was not statistically significant (*P* = 0.078), the long-term stability of LVIS SAC seemed better than that of laser-cut SAC for the treatment of wide-necked acutely RIA. The results were consistent with those reported by Ge et al. In that study, the author reported 96 cases of LVIS SAC and 112 cases of laser-cut SAC for unruptured intracranial aneurysms and found that the long-term complete occlusion rate was higher in the LVIS SAC group than in the laser-cut SAC group [28]. Wu, Yan, and Su W also confirmed the long-term efficacy of LVIS SAC for RIAs [36, 37, 39].

It is worth noting that high metal coverage may carry a high risk of ischemic complications [25]. A systematic review showed that the incidence of thromboembolic events in unruptured intracranial aneurysms treated with LVIS SAC was 4.9% [25]. Patients are considered to be in relatively hypercoagulable states in the acute phase of aneurysm rupture, and stent placement could synergistically trigger platelet aggregation. Therefore, we used a modified antiplatelet regimen in which a small dose of tirofiban was given in addition to a loading dose of aspirin (300 mg) and clopidogrel (300 mg) before stent release to prevent intraoperative thrombosis. A number of studies have confirmed the safety and efficacy of tirofiban in the treatment of RIAs [40–45]. Kim S et al. reported 40 cases of RIAs treated with SAC and intravenous administrations of tirofiban before stent release, and the results showed that intraoperative aneurysm rupture occurred in 2 cases (2.5%), but no thromboembolic events were observed [40]. Wang et al. compared the effects of tirofiban versus clopidogrel for preventing thrombus formation in RIAs treated with SAC and found that tirofiban significantly reduced the incidence of thromboembolic events compared with clopidogrel (3.91% vs 13.21%, *P* = 0.043) without increasing the risk for hemorrhagic events (2.34% vs 5.66%, *P* = 0.360) [42]. In the present study, the rates of hemorrhagic and ischemic complications in the LVIS stent group were 3.5% and 2.1%, respectively, which were lower than those reported previously [25, 29, 30, 46].

A large amount of literature has confirmed the safety and efficacy of flow diversion (FD) for the treatment of unruptured complex intracranial aneurysms, especially large, giant aneurysms, with favorable results on angiographic follow-up [47–49]. FDs are essentially low-porosity stents with a higher metal coverage than conventional stents and promote the reconstruction of the diseased vessel wall by diverting the flow away from the aneurysm sac and progressive intra-aneurysmal thrombosis [50–52]. Recently, the indications for FD have been extended to acutely RIAs, and the application of FD in blood blister-like aneurysms has shown preliminarily advantages in terms of safety and efficacy compared with SAC and overlapping stents [50, 53]. According to a recent meta-analysis of FD in the treatment of RIAs, FD for selective RIAs yielded high rates of long-term angiographic occlusion (90%) and favorable clinical outcomes (81%) [51]. Another systematic review reported by Cagnazzo F showed that the overall procedure-related complication rate of FD for RIAs was 17.8%, whereas the complication rate was higher in the posterior circulation than in the anterior circulation (27% vs 11.7%, *P* = 0.004) [50]. Most interestingly, the author noted that FD treatment for saccular RIAs was associated with a higher rate (23%) of complications than that for fusiform/dissecting RIAs (13%) and blood blister-like aneurysms (18%). In the present study, both LVIS stents and laser-cut SAC for saccular RIAs yielded lower perioperative procedural complication rates (5.6% and 14.0%, respectively) than the data above. Moreover, delayed stenosis and occlusion of the covered side branch, delayed aneurysm rebleeding and symptomatic cerebral infarction cannot be neglected for patients treated with FD [50, 54–56]. In contrast, none of the patients in this study showed delayed occlusion/stenosis of the covered side branch and delayed aneurysm rebleeding during the follow-up period. Taking these studies and the present results into account, LVIS SAC of saccular RIAs is superior to FD in terms of perioperative safety, but longer follow-up studies are needed to further evaluate the long-term efficacy.

The present study is the first cohort study to compare the safety and efficacy of LVIS stent-assisted coil embolization and laser-cut stent-assisted coil embolization in the treatment of wide-necked acutely RIAs. The retrospective design is the biggest limitation of this study; thus, missing data and case selection bias were difficult to avoid. In addition, the sample size in this study was relatively small, and some differences may not be detected. For example, the angiographic follow-up results revealed that the LVIS stent group had a higher complete occlusion rate and lower recurrence rate than the laser-cut stent group, but the difference was not statistically significant (*P* = 0.078), since *P* values < 0.05 were considered statistically significant. However, we believe that with an increased sample size, the differences in complete occlusion rate and recurrence rate between LVIS SAC and laser-cut SAC in the treatment of wide-necked acutely RIA would become significant, and the advantage in terms of long-term efficacy of LVIS stents would also emerge.

## Conclusions

LVIS stent-assisted coil embolization can reduce the incidence of intraprocedural rupture and overall procedure-related complications compared with laser-cut stent-assisted coil embolization for the treatment of wide-necked acutely RIAs. Prospective studies with larger sample sizes are needed to further confirm the safety and efficacy of this strategy.

## Data Availability

The datasets used and analyzed during the current study are available from the corresponding author on reasonable request.

## Abbreviations

SAC: Stent-assisted coiling
RIA: Ruptured intracranial aneurysms
LVIS: Low-profile visualized intraluminal support
CT: Computed tomography
DSA: Digital subtraction angiography
